# Dissociations within short-term memory in GluA1 AMPA receptor subunit knockout mice

**DOI:** 10.1016/j.bbr.2011.05.016

**Published:** 2011-10-10

**Authors:** Amy M. Taylor, Burkhard Niewoehner, Peter H. Seeburg, Rolf Sprengel, J. Nicholas P. Rawlins, David M. Bannerman, David J. Sanderson

**Affiliations:** a Department of Experimental Psychology, University of Oxford, South Parks Road, Oxford OX1 3UD, UK; b Max-Planck Institute of Medical Research, Department of Molecular Neurobiology, Jahnstrasse 29, D-69120 Heidelberg, Germany

**Keywords:** AMPA receptors, Conditional learning, Trace conditioning, Non-spatial memory, Spatial memory

## Abstract

GluA1 AMPA receptor subunit knockout mice display a selective impairment on short-term recognition memory tasks. In this study we tested whether GluA1 is important for short-term memory that is necessary for bridging the discontiguity between cues in trace conditioning. GluA1 knockout mice were not impaired at using short-term memory traces of T-maze floor inserts, made of different materials, to bridge the temporal gap between conditioned stimuli and reinforcement during appetitive discrimination tasks. Thus, different aspects of short-term memory are differentially sensitive to GluA1 deletion. This dissociation may reflect processing of qualitatively different short-term memory traces. Memory that results in performance of short-term recognition (e.g. for objects or places) may be different from the memory required for associative learning in trace conditioning.

## Highlights

► GluA1 AMPAR subunit is important for short-term recognition memory. ► We tested whether GluA1 is necessary for short-term memory in trace conditioning. ► GluA1 deletion impaired rewarded alternation, but not trace conditioning. ► The dissociation may reflect processing of different short-term memory traces.

Genetically modified mice lacking the GluA1 AMPA receptor subunit (henceforth referred to as GluA1^−/−^ mice [1]) display impaired short-term memory. For example, GluA1^−/−^ mice fail to demonstrate short-term memory for recently visited spatial locations [4,5,11,3,9], or recently experienced objects [7] when assessed using novelty preference tests. GluA1 deletion also affects the expression of short-term memory for recently presented, visual stimuli in an operant chamber [8]. In contrast, long-term memory is intact [11,3,9,7] or even enhanced [5] in GluA1^−/−^ mice. Thus, GluA1 plays a specific role in short-term memory.

In trace conditioning a short-term memory trace of the conditioned stimulus (CS) is required to bridge the interval or discontiguity between the CS and the unconditioned stimulus (US). It is possible that GluA1 is necessary for short-term maintenance of memory that is necessary for trace conditioning as well as short-term memory required for discriminating between stimuli on the basis of how recently they have been experienced.

There is some evidence to suggest that GluA1 may be important for maintaining memory traces in conditional discriminations. We have previously tested GluA1^−/−^ mice on a T-maze task in which floor inserts made from different materials (A and B) present in the start arm acted as conditional cues, indicating whether the left or right goal arm (L and R) was rewarded [10; e.g. A: L+R−, B: L−R+]. GluA1^−/−^ mice were able to acquire the task if the floor inserts extended throughout the entire maze, including the start arm and both goal arms, but not if the floor inserts were only present in the start arm of the maze. Thus the absence of the conditional cue (the floor insert) at the time when the place-reward association was experienced determined whether or not the mice were impaired. This may suggest an important role for GluA1 in short-term memory for stimuli acting as conditional cues.

To test whether GluA1 is necessary for trace conditioning we first trained mice on a simple discrimination task in which different floor inserts signalled whether food was present or absent in a particular goal arm of a T-maze (Experiment 1). Task difficulty was manipulated by increasing the temporal and spatial discontiguity between the floor inserts and the presence/absence of food reward. We then tested mice on non-spatial versions of the conditional T-maze task, either with or without a discontiguity between the conditional cues and the goal arm cues (Experiments 2 and 3).

## 1 Methods

### 1.1 Subjects

Experimentally naïve, littermate, age-matched female, wild-type (WT) and GluA1^−/−^ mice, bred in the Department of Experimental Psychology at the University of Oxford, served as subjects in the following experiments [see 13, for details of genetic construction, breeding and subsequent genotyping]. Mice were housed in group cages and tested during the light phase of the day (0700–1900). They had ad libitum access to water but were maintained on a restricted feeding schedule at 85% of their free-feeding weight throughout behavioral testing. All experiments were conducted under the auspices of UK. Home Office Project and Personal licences held by the authors.

### 1.2 Apparatus

Experiment 1 (Simple trace conditioning) was conducted in a grey-painted, elevated, wooden T-maze that consisted of a start arm (47 cm × 10 cm) and two identical goal arms (35 cm × 10 cm) surrounded by a 10 cm high wall. A metal food well was located 3 cm from the end of each goal arm. A pair of grey-painted wooden guillotine doors was present in each goal arm (at a distance of 10 and 20 cm from the entrance to the goal arm), creating an antechamber between the floor insert and the food well in which the mouse could be contained and a delay imposed between the discriminanda and the presence/absence of reward. A clear Perspex cover was placed over each antechamber to prevent the mice climbing out. Wooden floor inserts (9.5 cm × 9.5 cm) covered with different materials acted as discriminanda signalling the location of the reward (Experiment 1A—blue towel vs. black foam rubber; Experiment 1B—green plastic circles vs. orange sandpaper or white Perspex vs. wire mesh). Inserts were placed at the entrance to each goal arm, between the choice point and the first guillotine door.

Experiment 2 (non-spatial, conditional learning, contiguous version) and Experiment 3 (non-spatial, conditioning learning, discontiguous version) were conducted in cross-maze of similar construction, which had two start arms (North and South; 47 cm × 10 cm) and two goal arms (East and West; 35 cm × 10 cm). The entrance to one of the start arms was always blocked (the North arm), thus creating a T-maze similar to that used in Experiment 1. In addition, each goal arm was blocked off 10 cm from the choice point using a wooden grey block to create goal arms that were 10 cm long. Floor inserts of different lengths, made from either white Perspex or wire mesh (5 mm × 5 mm mesh, affixed to a grey painted, wooden backing), could be placed into the start arm of the maze to act as conditional cues. A second set of floor inserts (width × length: 9.4 cm × 10 cm), made from either cream-coloured sandpaper or green plastic circles (diameter 15 mm), were placed in the goal arms. A metal food well assembly was located at the extreme far end of each of the goal arm inserts. This consisted of a small metal food well (diameter 1 cm; height 0.5 cm), which contained sweetened condensed milk (diluted 50/50 with water). The small well was itself contained in a larger hexagonal well (diameter 2.2 cm; height 1.1 cm) that also contained sweetened condensed milk to act as an odour mask. Access to the milk in the larger, hexagonal well was prevented by a fine wire mesh. The overall food well assembly was inclined at a slight angle, facing away from the choice point, to ensure that the animals could not see the presence/absence of reward in the food wells. A thin metal bar was positioned 1 cm above the floor and 1.2 cm from the choice point in each of the goal arms. Entry into an arm was defined as when the mouse had placed both of its forepaws over the barrier and into the goal arm.

### 1.3 Procedure

#### 1.3.1 Habituation phase

Mice were first habituated to drinking sweetened, condensed milk (diluted 50:50 with water) in their home cages. They were then habituated over several days to the maze in which the experiment was to be conducted until they were running freely and readily consuming the reward.

#### 1.3.2 Experiment 1A—simple trace conditioning

**Fig. 1:**
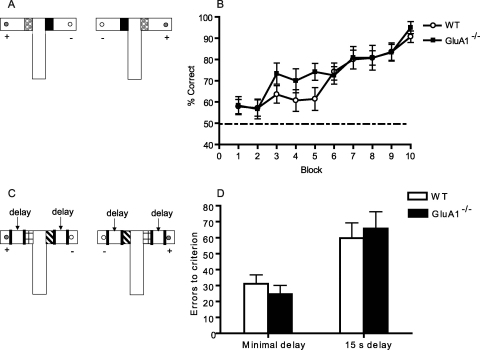
*Experiment 1*: Simple appetitive trace conditioning is unaffected by GluA1 deletion. (A) Experimental design. WT and GluA1^−/−^ mice were required to learn which of two floor inserts was associated with reward. There was a discontiguity between the discriminanda and the presence/absence of reward. (B) Mean ± S.E.M. percent correct choices per block of 10 trials for WT and GluA1^−/−^ mice. Dashed line equals chance level of performance (50%). (C) Experimental design with an additional 15 s delay between floor insert stimuli and reward/non-reward. (D) Mean ± S.E.M. errors to obtain a criterion score of at least 17 correct choices out of 20 trials over 2 consecutive days of testing for WT and GluA1^−/−^ mice, with either minimal delay (left) or an additional delay of 15 s (right). Mice received 10 trials per day for 10 days with an ITI of approximately 5–10 min throughout Experiment 1.

Experimentally naïve WT (*N* = 14) and GluA1^−/−^ (*N* = 12) mice were first trained to discriminate between a blue towel insert and black foam rubber insert, with a spatiotemporal discontiguity between the discriminanda and the presence/absence of reward (Fig. 1A). Mice were assigned to either towel or foam insert groups which signified which cue was associated with reward, and this was counterbalanced with respect to group, such that equal numbers of knockout and wildtype animals were trained to each of the inserts. Animals were rewarded with 0.1 ml of sweetened condensed milk (diluted 50/50 with water) for choosing the correct insert. If the mouse chose the incorrect insert, it was removed from the maze immediately without receiving reward. A choice was defined as when a mouse had placed all four paws onto an insert. The goal arm (left/right) in which the correct insert was placed for each trial was determined by a pseudorandom sequence (with equal numbers of placements in the two arms in any one session, and no more than three consecutive trials to the same arm).

On day 1 of testing, each animal received 6 forced trials to each of the rewarded and non-rewarded insert arms prior to commencement of free-choice testing in order to habituate them to the inserts and overcome any innate aversion to either of the cues. During these forced trials the mouse was forced into one of the goal arms by the presence of a grey-painted wooden block in the opposite arm, preventing entry into that arm. The mice then received reward or no reward according to their allocations. On choice trials the animal was placed in the start arm facing the wall and allowed to enter one of the goal arms. Animals received 10 trials per day for 10 days with an ITI of approximately 5–10 min.

#### 1.3.3 Experiment 1B—simple trace conditioning with an additional delay

The memory demands of the simple trace conditioning task were increased by adding a 15 s delay being the insert cues and the presence/absence of reward (Fig. 1C). This was achieved by holding the mouse in the antechamber between the pair of guillotine doors for 15 s. Mice previously trained in Experiment 1A were trained on the trace conditioning task with the additional delay using new pairs of floor inserts. The mice were first habituated to experiencing a delay, using the previously experienced towel/foam inserts (Experiment 1A). They received 10 trials per day for 3 days. On the first day they were contained in the antechamber, between the floor insert and the food well, for 5 s prior to obtaining the reward, on the second day for 10 s and on the third day for 15 s.

The mice were then assigned to two new insert pairs: (i) sandpaper vs. plastic circles and (ii) wire mesh vs. Perspex. One insert pair was associated with an additional delay (15 s delay vs. no delay). This was counterbalanced such that approximately 25% of the animals began the experiment with a delay-sandpaper vs. circles task, 25% with no delay-sandpaper vs. circles, 25% with delay-wire vs. Perspex and 25% with no delay-wire vs. Perspex tasks. It was also counterbalanced with respect to which insert was rewarded in each pair. The animals then received 6 forced trials to each of the rewarded and non-rewarded insert arms, with delay or no delay included as appropriate.

The animals were placed in the start arm facing the end wall and allowed to choose one of the two inserts placed just inside the goal arm. The goal arm (left/right) in which the rewarded insert was placed was determined for each trial by a pseudorandom sequence (with equal numbers of placements in the two arms in any one session, and no more than three consecutive trials to the same arm). The guillotine door nearest the food well was closed and the door nearest the start arm was open at the start of the trial. Animals assigned to the delay condition were retained in the antechamber for 15 s before the door nearest the food well was raised and they were permitted access to the reward (if they had chosen correctly). Animals choosing the non-rewarded insert were also contained for 15 s. For animals assigned to the no delay condition the door nearest the reward was raised immediately after choosing.

Animals received 10 trials per day until they reached a criterion of 17 or more out of 20, across 2 consecutive days of testing. Once the animal had achieved criterion, it received a further day of testing during which the correct insert/arm was baited only after the animal had made its choice (post-choice baiting).

The animal was then assigned a second task, but with a new pair of floor inserts and the opposite delay/no-delay condition. Thus, the effect of additional delay on trace conditioning was assessed using a within-subjects design. For example, if a given mouse was first trained on a delay-wire mesh vs. Perspex problem, it would then be assigned a no delay-sandpaper vs. plastic circles problem. The mouse again first received 6 forced trials to each of the new inserts prior to free choice testing. It was then trained until reaching the same criterion of 17 or more out of 20, across 2 consecutive days of testing, prior to a single test session of 10 post-choice baiting trials.

#### 1.3.4 Experiment 2—non-spatial, conditional learning, contiguous-version

**Fig. 2:**
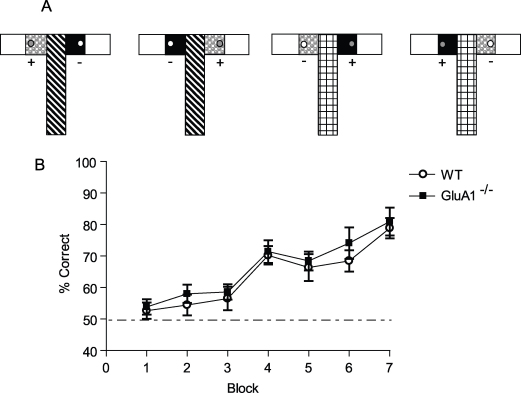
*Experiment 2*: Non-spatial, conditional discrimination (contiguous version) is unaffected by GluA1 deletion. (A) Experimental design. Mice were trained on a conditional learning task in which a floor insert (e.g. Perspex vs. wire mesh), covering the whole of the start arm acted as a conditional cue indicating which of two goal arm inserts (e.g. sandpaper vs. plastic circles) was associated with a milk reward. (B) Mean ± S.E.M. percent correct choices per block of 48 trials for WT and GluA1^−/−^ mice. Dashed line equals chance level of performance (50%). Mice received 12 trials per day for 28 days with an ITI of approximately 5–10 min throughout Experiment 2.

Experimentally naïve WT (*N* = 7) and GluA1^−/−^ mice (*N* = 7) mice were trained on a conditional learning task in which a floor insert (Perspex vs. wire mesh, 57 cm × 10 cm), covering the whole of the start arm and extending right across to the wall opposite the start arm at the junction of the maze, acted as a conditional cue indicating which of two goal arm inserts (sandpaper vs. plastic circles) was associated with a milk reward. Thus, in this experiment the start arm insert was contiguous with both of the goal arm inserts (Fig. 2A). For half of the mice the presence of the Perspex insert indicated that the 0.1 ml milk reward was available in the goal arm containing the sandpaper floor insert. In contrast, the reward was in the plastic circles insert/goal arm if the start arm contained the wire mesh, floor insert. For the remaining mice, the opposite pair of start arm/goal arm insert/reward contingencies applied (e.g. Perspex/plastic circles, wire mesh/sandpaper). The relationships between the floor inserts in the start arm and the rewarded/non-rewarded goal arm inserts were constant for each animal throughout the experiment. Mice received 28 sessions comprising 12 trials per session with an ITI of 5–10 min. Each session consisted of equal numbers of trials with each of the two start arm floor inserts, and no more than 3 consecutive trials with the same start arm insert, according to a pseudorandom sequence. The left/right orientation of the sandpaper and plastic circles goal arm inserts also varied from trial to trial, according to another, superimposed pseudorandom sequence, with the reward being in the left or right goal arm on an equal numbers of trials, and with no more than 3 left or right correct choices in a row. At the start of each trial, the mouse was placed into the start arm at the end furthest from the choice point, and allowed a free choice of either goal arm. A correct choice was rewarded with 0.1 ml of milk. If the mouse chose incorrectly, it was immediately removed form the maze without any reward.

##### 1.3.4.1 Probe sessions—cued vs. non-cued trials

Once the mice had acquired the task a set of probe trials (2 blocks of 12 trials each) was conducted to determine whether the mice were able to solve the task by seeing or smelling the milk rewards from the choice point. Normal “cued” trials in which the start arm floor insert was present were interleaved with “non-cued” trials in which no start arm insert was present. Cued trials were run according to the same procedure as during training with the full-length start arm insert present. For non-cued trials there was no start arm insert present, only goal arm inserts, and the milk reward was allocated to one of the two goal arms according to a pseudorandom sequence. The absence of the conditional cue renders the task insoluble and performance should fall to chance. Cued and non-cued trials were interleaved according to a pseudorandom sequence with no more than 3 trials of each condition in a row. The left/right position of the goal arms inserts and the location of the reward were also fully counterbalanced across each block of 12 trials (each block contained 6 cued and 6 non-cued trials).

##### 1.3.4.2 Probe sessions—reducing the length of the start arm insert

**Fig. 3:**
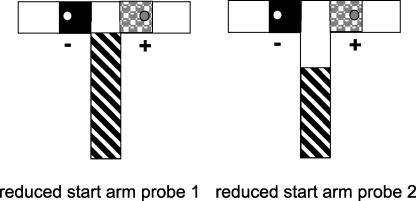
Experimental design for probe trials in which the length of the start arm floor insert conditional cue was reduced, after mice had already acquired the task (Experiment 2). First, the length of the start arm insert was reduced to 47 cm, such that it stopped at beginning of the choice area (reduced start arm probe 1). Then the length of the start arm insert was further reduced to 35 cm, and now covered approximately 60% of the start arm (reduced start arm probe 2). This resulted in a 12 cm discontiguity between the start arm insert and goal arm inserts.

In a second set of probe sessions, a discontiguity was introduced between the start arm insert and the goal arm inserts by reducing the length of the start arm insert (Fig. 3). First, the length of the start arm insert was reduced to 47 cm, such that it stopped at beginning of the choice area (reduced start arm probe 1, Fig. 3). Otherwise, the experimental procedure was the same as during training, and mice received a total of 48 trials with this condition. Then the length of the start arm insert was further reduced to 35 cm, and now covered approximately 60% of the start arm (reduced start arm probe 2, Fig. 3). This resulted in a 12 cm discontiguity between the start arm insert and goal arm inserts. Mice received 24 trials in total with this arrangement.

### 1.4 Experiment 3—non-spatial, conditional learning–discontiguous version

**Fig. 4:**
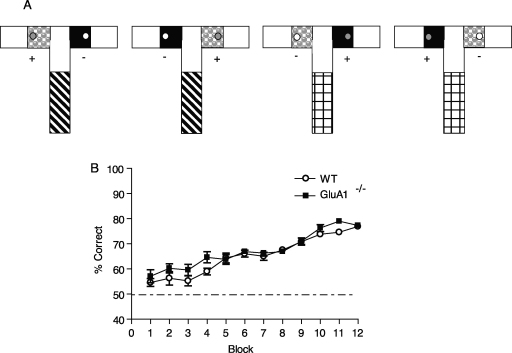
*Experiment 3*. Non-spatial, conditional discrimination (discontiguous version) is enhanced by GluA1 deletion. (A) Experimental design. Separate groups of mice were trained on the conditional learning task, but now using a shorter start arm floor insert during acquisition of the task. This start arm insert now covered approximately 60% of the start arm and resulted in a 12 cm discontiguity between the start arm and goal arm floor coverings. Otherwise the testing protocol was the same as for Experiment 2. (B) Mean ± S.E.M. percent correct choices per block of 48 trials for WT and GluA1^−/−^ mice. Dashed line equals chance level of performance (50%). Mice received 16 trials per day for 36 days with an ITI of approximately 5–10 min throughout Experiment 2.

Experimentally naïve WT (*N* = 10) and GluA1^−/−^ mice (*N* = 10) mice were trained on the same conditional learning task, but now using the shorter start arm floor insert (35 cm × 10 cm), during acquisition of the task. This start arm insert covered approximately 60% of the start arm and resulted in a 12 cm discontiguity between the start arm and goal arm floor coverings (Fig. 4A). Otherwise the testing protocol was the same as for Experiment 2.

#### 1.4.1.1 Probe trials—cued vs. non-cued trials

A set of cued and non-cued probe trials (two blocks of 16 trials/block) were conducted, as in Experiment 2, to determine whether the mice were able to solve the task by seeing or smelling the milk rewards from the choice point. Normal “cued” trials in which the start arm floor insert was present were interleaved with “non-cued” trials in which no start arm insert was present.

#### 1.4.1.2 Probe trials—neutral goal arms inserts

A final set of probe trials (four blocks of 16 trials/block) was conducted to determine whether the mice acquired the task by learning to approach the rewarded goal arm insert in the presence of a particular start arm insert, or to avoid the unrewarded goal arm insert in the presence of a given start arm cue, or by adopting a combination of both stratagems. Either the rewarded or non-rewarded goal arm insert was replaced by a neutral, goal arm insert, of the same dimensions, and made from grey foam rubber. The mice had no prior exposure to this insert. Within each block of 16 trials, 8 trials were run as normal trials with both the rewarded and non-rewarded inserts in place, as during training. Eight probe trials were interleaved with the normal training trials, according to a pseudorandom sequence. Furthermore, for the 8 probe trials in each block, the neutral insert replaced either the rewarded or non-rewarded goal arm insert on an equal number of trials, again according to a pseudorandom sequence. The left/right location of the neutral insert was also fully counterbalanced across each block of 16 trials.

### 1.5 Spatial working memory testing

All mice from Experiments 1–3 were tested on spatial working memory (non-matching to place; NMTP [3]). Mice received 20 trials of discrete trial, rewarded alternation testing using the same elevated T-maze but with no floor inserts present. Each trial consisted of a sample run and a choice run. On the sample run the mice were forced either left or right by a wooden block to obtain a milk reward, according to a pseudorandom sequence (with equal numbers of left and right turns per session, and with no more than 3 consecutive turns in the same direction). The block was then removed and the mouse placed, facing the experimenter, at the end of the start arm and allowed a free choice of either arm. The time interval between the sample run and the choice run was approximately 15 s. The mouse was rewarded for choosing the previously unvisited arm (i.e. for alternating).

## 2 Results

### 2.1 Experiment 1A—simple trace conditioning

Both WT and GluA1^−/−^ mice acquired the simple, appetitive, trace discrimination, and at an equivalent rate (effect of block, *F*(9,216) = 21.6; *p* < 0.0001; no significant effect of group, *F* < 1, nor group by block interaction, *F*(9,216) = 1.1; *p* > 0.20; Fig. 1B).

### 2.2 Experiment 1B—simple trace conditioning with an additional delay

When the delay between the floor inserts and the presence/absence of reward was increased, mice took longer to acquire the simple discriminations (effect of delay, *F*(1,24) = 12.5; *p* < 0.002; Fig. 1D). However, the WT and GluA1^−/−^ groups were still indistinguishable (effect of genotype and delay by genotype interaction, *F* values < 1). Mice performed significantly above chance on the post-choice baiting sessions, confirming that the mice were not solving the task by smelling the rewards (minimal delay: WT, 85.7 ± 2% S.E.M., GluA1^−/−^, 90.0 ± 1.7% S.E.M.; 15 s delay: WT, 84.3 ± 1.4% S.E.M., GluA1^−/−^, 83.3 ± 1.4% S.E.M., all one-sample *t*-test *p* values < 0.0005, effect of group, *F* < 1).

#### 2.2.1 Spatial working memory

**Fig. 5:**
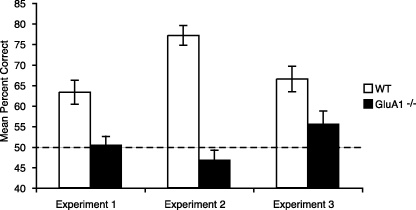
Spatial working memory is impaired by GluA1 deletion. Rewarded alternation performance in WT and GluA1^−/−^ mice in Experiments 1–3. Mice received 20 trials of rewarded alternation. The mean percentages of correct alternation responses (±S.E.M.) are shown. The dashed line indicates chance performance.

On completion of the trace memory study, all mice received 20 trials of rewarded alternation (spatial working memory) testing. In agreement with previous research [3], GluA1^−/−^ mice were impaired relative to their WT controls (*t*(24) = 3.5; *p* < 0.002, Fig. 5).

### 2.3 Experiment 2—non-spatial, conditional learning, contiguous-version

Both WT and GluA1^−/−^ mice acquired the contiguous version of the non-spatial conditional task (Fig. 2B). There were no differences between the groups (effect of group and group by block interaction, both *F* values < 1; effect of block, *F*(6,72) = 22.4; *p* < 0.001).

#### 2.3.1 Probe sessions—cued vs. non-cued trials

**Fig. 6:**
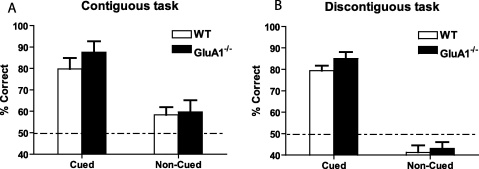
Performance of WT and GluA1^−/−^ mice on cued vs. non-cued probe trials. Mean ± S.E.M. percent correct choices on normal trials (cued) and on trials during which the start arm conditional cue was removed. Under these conditions the task should be insoluble. Dashed line equals chance level of performance (50%). (A) Contiguous, non-spatial version of the task (Experiment 2). (B) Discontiguous, non-spatial version of the task (Experiment 3).

The data for one GluA1^−/−^ mouse was lost for the probe sessions and for the subsequent spatial working memory testing. On probe trials during which the start arm conditional cue floor insert was absent from the maze, performance of both WT and GluA1^−/−^ mice fell to near-chance levels (Fig. 6A). In contrast, performance on the inter-leaved trials, during which the start arm insert was still present (as during training), remained high in both groups. Analysis of variance (ANOVA) revealed a highly significant main effect of trial-type (cued vs. non-cued; *F*(1,11) = 65.1; *p* < 0.001), but neither a main effect of group (*F* < 1), nor a group by trial-type interaction (*F*(1,11) = 1.1; *p* > 0.20). Both groups failed to perform significantly above chance when the conditional cue was absent (WT, *t*(6) = 2.3; GluA1^−/−^, *t*(5) = 1.8, *p* values > 0.05).

#### 2.3.2 Probe sessions—reducing the length of the start arm insert

Reducing the length of the start arm insert to 47 cm such that it stopped at the beginning of the choice area (Fig. 3, left) had little, if any effect on choice accuracy. Both WT (80.4 ± 3.1% correct) and GluA1^−/−^ mice (82.6 ± 4.3% correct) maintained a high level of performance and there was no group difference (*t* < 1). However, a further reduction in the length of the start arm (35 cm), such that there was now a 12 cm discontiguity between the conditional cue and the goal arm inserts (Fig. 3, right), resulted in a dramatic drop in performance to chance levels in both the wild type (54.8 ± 1.9% correct) and GluA1^−/−^ mice (55.6 ± 2.3% correct), although again there was no group difference (*t* < 1).

#### 2.3.3 Spatial working memory

On completion of this non-spatial conditional task, 20 trials of rewarded alternation testing revealed a spatial working memory deficit in the GluA1^−/−^ mice relative to WT controls (*t*(11) = 8.8; *p* < 0.001, Fig. 5).

### 2.4 Experiment 3—non-spatial, conditional learning, discontiguous version

Both WT and GluA1^−/−^ mice acquired the discontiguous, non-spatial conditional task (Fig. 4B). However, the performance of the knockout mice was slightly superior to that of the controls across training (overall performance: WT, 65.3% correct ± 0.8 S.E.M., GluA1^−/−^, 67.4% correct ± 0.5 S.E.M; effect of genotype, *F*(1,18) = 5.34, *p* < 0.05; effect of block, *F*(11,198) = 45.13, *p* < 0.001; block by genotype interaction, *F* < 1).

#### 2.4.1 Probe trials—cued vs. non-cued trials

Performance fell slightly below chance level in both groups of mice (WT, *t*(9) = 2.7, *p* < 0.03; GluA1^−/−^, *t*(9) = 2.3, *p* < 0.05) when there was no floor insert present in the start arm, confirming that mice are solving the task by attending to the maze inserts rather than seeing or smelling the rewards (Fig. 6B). ANOVA confirmed that there was a highly significant main effect of trial type (cued vs. non-cued *F*(1,18) = 182.0; *p* < 0.001). There was no main effect of group (*F*(1,18) = 1.7; *p* > 0.20), nor any group by trial type interaction (*F* < 1).

#### 2.4.2 Probe trials—neutral goal arms inserts

**Fig. 7:**
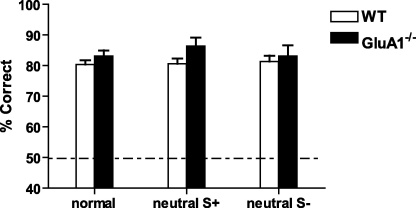
Performance of WT and GluA1^−/−^ mice on probe trials during which either the S+ or S− goal arm inserts were removed and replaced by a neutral insert after acquisition of the discontiguous, non-spatial version of the task (Experiment 3). Mean ± S.E.M. percent correct choices on normal trials (normal) and on trials during which either the S+ (neutral S+) or the S− (neutral S−) insert was replaced. Dashed line equals chance level of performance (50%).

Replacing either the “to be rewarded; S+” goal arm insert or the “not to be rewarded, S−” goal arm insert with a neutral insert had no effect on performance in any of the groups of mice. GluA1^−/−^ and WT mice maintained a high level of performance on all trial types (Fig. 7). ANOVA indicated that there was no effect of trial type (normal vs. S+ replaced vs. S− replaced; *F* < 1), no effect of group (*F*(1,18) = 1.5; *p* > 0.20), and no group by trial type interaction (*F* < 1).

#### 2.4.3 Spatial working memory

The GluA1^−/−^ mice exhibited a spatial working memory deficit during T-maze rewarded alternation testing (*t*(18) = 2.5; *p* < 0.025, Fig. 5).

## 3 Discussion

The results show that GluA1^−/−^ mice were not impaired on trace conditioning tasks in which they had to maintain a short-term memory of floor inserts made of different materials in order to obtain reward. They successfully acquired a simple discrimination, with and without additional delays (Experiment 1), and a non-spatial, conditional discrimination, with and without a discontiguity between the start arm conditional cue and the goal arm cues (Experiments 3 and 2, respectively). This contrasts with the impairments in both spatial and non-spatial, short-term recognition memory displayed by GluA1^−/−^ mice [4,5,7,8].

The successful acquisition of the discontiguous, non-spatial conditional task is also in contrast to the impairment seen on the spatial version in which a start arm floor insert indicated whether the left or right goal arm was rewarded [10]. This dissociation shows that these tasks are supported by different neurobiological mechanisms, and suggests that they may be solved in different ways. It is possible the dissociation is caused by the different types of information used (i.e. spatial/nonspatial) in the conditional tasks. However, the short-term memory deficit is not specific to spatial information in GluA1^−/−^ mice [7,8]. Furthermore, whilst GluA1 deletion impairs some spatial tasks such as spatial working memory [3,9] and short-term spatial recognition memory [4,5], it also spares spatial reference memory [3,9] and even enhances long-term spatial recognition memory [5]. This may imply that it is unlikely that the dissociation between the tasks is simply due to the information content. Another possibility is that the tasks differ in the type of psychological operation being performed. For example, it is possible that the start arm insert acts as an occasion setter, modulating the associative strength of the goal arm inserts, or alternatively, the discrimination may be solved by the start and goal arm inserts forming distinct configural cues [e.g. 2]. The validity of these different accounts requires further investigation.

Our data suggest that different aspects of short-term memory are differentially sensitive to GluA1 deletion. It is possible that different short-term memory traces are necessary for trace conditioning and recency-dependent recognition. Wagner [12] suggested that a stimulus presentation leads to activation of a mnemonic representation in two different memory states, A1 and A2. Whereas trace conditioning may rely on the A1 state memory, short-term recognition, as indicated by novelty preference, may rely on A2 state memory.

**Fig. 8:**
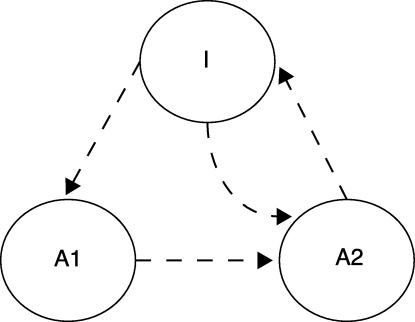
The states of activation, which stimulus representations can reside, and the permissible transitional routes between states, according to Wagner [12]. When a stimulus is presented its representation increasingly enters the A1 state from the inactive state (I). From the A1 state representations rapidly decay to the A2 state before eventually returning to the inactive state. Short-term recognition memory is dependent on the strength of activation in the A2 state, whereas trace conditioning is dependent on the strength of activation in the A1 state (see main text). Associative retrieval of information into short-term memory is permissible by the route from the I to A2.

According to Wagner [12] when a stimulus is presented a representation of the stimulus increasingly enters a primary activity state (A1) before decaying into a secondary activity state (A2) where it remains before eventually returning to an inactive state (I, Fig. 8). The temporal dynamics of the transitions betweens these different memory states determine the extent of short-term recognition memory and the extent of trace conditioning. In the case of short-term recognition a recently presented stimulus’ representation will have decayed to the A2 when the stimulus is subsequently presented after a short-interval. A2 state representations cannot return to the A1 state when a stimulus is re-presented. They are also less able to activate responding than representations active in the A1 state. A consequence of this is that a recently experienced stimulus will elicit less exploration than a novel or less recently experienced stimulus (i.e. novelty preference). If, as we have previously proposed [6], GluA1 deletion reduces the rate at which representations transfer to from the A1 state to the A2 state then the A2 state memory, caused as a result of a recent stimulus presentation, would be weaker than in GluA1^−/−^ mice than in control mice, resulting in impaired short-term recognition memory [4,5,7,8].

In the case of trace conditioning the strength of associative learning is dependent on the strength of stimulus representations in A1. Stimuli whose representations are coactive in A1 are able to form excitatory associations. Therefore, if an interval is placed in between the CS and US such that the CS's representation has partially decayed to the A2 state when the US is presented, then the extent of associative learning that can take place is reduced. If, as mentioned previously, GluA1 deletion reduces the rate of at which A1 state representations transfer to the A2 state then it would not be predicted that GluA1 deletion would impair trace conditioning. The results of Experiments 1–3 are consistent with this prediction. Furthermore, a reduction in the rate of transfer from the A1 state to the A2 state in GluA1^−/−^ mice may lead to actually enhancing trace conditioning, relative to control mice, due to increasing the strength of the A1 representation at the time at which the US is presented. This prediction is consistent with the results of Experiment 3 in which learning was enhanced in GluA1^−/−^ mice when there was a discontiguity between the cues that predicted food reward. At present, we cannot rule out the possibility that this enhancement reflects non-specific effects such as differences in running speeds between groups. Thus, further studies are required to ascertain whether conditions can be found under which trace memory is facilitated in GluA1^−/−^ mice.
